# *Itm2a*, a Target Gene of GATA-3, Plays a Minimal Role in Regulating the Development and Function of T Cells

**DOI:** 10.1371/journal.pone.0096535

**Published:** 2014-05-15

**Authors:** Tzong-Shyuan Tai, Sung-Yun Pai, I-Cheng Ho

**Affiliations:** 1 Division of Rheumatology, Immunology, and Allergy, Department of Medicine, Brigham and Women's Hospital, Boston, Massachusetts, United States of America; 2 Harvard Medical School, Boston, Massachusetts, United States of America; 3 Department of Medical Research, E-Da Hospital, I-Shou University, Kaohsiung, Taiwan; 4 Division of Pediatric Hematology-Oncology, Department of Pediatrics, Children's Hospital Boston, Boston, Massachusetts, United States of America; 5 Department of Pediatric Oncology, Dana-Farber Cancer Institute, Boston, Massachusetts, United States of America; University of Alberta, Canada

## Abstract

The integral membrane protein 2a (Itm2a) is one of the BRICHOS domain-containing proteins and is structurally related to Itm2b and Itm2c. It is expressed preferentially in the T lineage among hematopoietic cells and is induced by MHC-mediated positive selection. However, its transcriptional regulation and function are poorly understood. Here we showed Itm2a to be a target gene of GATA-3, a T cell-specific transcription factor. Deficiency of Itm2a had little impact on the development and function of polyclonal T cells but resulted in a partial defect in the development of thymocytes bearing a MHC class I-restricted TCR, OT-I. In addition, Itm2a-deficient mice displayed an attenuated T helper cell-dependent immune response in vivo. We further demonstrated that Itm2b but not Itm2c was also expressed in T cells, and was induced upon activation, albeit following a kinetic different from that of Itm2a. Thus, functional redundancy between Itm2a and Itm2b may explain the minimal phenotype of Itm2a deficiency.

## Introduction

GATA-3 is the third member of the GATA family of transcription factors [1]. Among hematopoietic cells, GATA-3 is expressed nearly exclusively in the T lineage, and is critical for the development of CD4^+^ T helper (Th) cells [2], the differentiation of T helper type 2 (Th2) cells [3], [4], the function of regulatory T (Treg) cells [5], [6], and the activation/homeostasis of CD8^+^ cytolytic T (Tc) cells [7], [8]. However, it is still not fully understood how GATA-3 executes its function. Several GATA-3 targets genes have been identified. For example, GATA-3 directly regulates the expression of Th-POK (encoded by *Zbtb7b*) [9], a transcription factor that is also essential for the development of CD4^+^ T cells [10], [11]. However, reconstitution of Th-POK was unable to rescue the development of GATA-3-deficient CD4^+^ T cells, suggesting the presence of additional GATA-3 target genes in mediating its function.

Itm2a is a type II transmembrane protein and contains 263 amino acid residues [12]. It is highly conserved between human and mouse. Itm2a belongs to the Type II Integral Membrane protein (Itm2) family, which also includes Itm2b and Itm2c [13]. Members of this protein family contain a BRICHOS domain, which possesses chaperone activity [14], a transmembrane domain at the N-terminus, and a furin cleavage site at the C-terminus. Among the Itm2 family members, Itm2b is the most extensively studied protein. The ectodomain of Itm2b can undergo at least three steps of protease-mediated cleavage [15], [16]. The first cleavage occurs at the C-terminus and is mediated by furin. The remaining Itm2b protein can be further cleaved by ADAM10 protease, releasing the C-terminal half, including the BRICHOS domain, into extracellular compartments. The remaining N-terminal half of Itm2b can then undergo regulated intramembrane proteolysis mediated by signal peptide peptidase (SPP) 2a and 2b. This intramembrane proteolytic event yields an intracellular domain (ICD) and a secreted low molecular weight peptide. Two types of mutation of Itm2b have been link to familial British Dementia (FBD) and familial Danish Dementia (FDD) [17], [18]. Both mutations result in the addition of 10 amino acid residues to the furin-cleaved C-terminal peptide, which accumulates in CNS forming the pathogenic plaques found in these patients. However, how the addition of 10 amino acids leads to the development of dementia is still unclear. Given the high degree of similarity in protein structure among itm2 proteins, it is very likely that such protease-mediated cleavages also occur on Itm2a. Evidence supporting this notion however is still lacking.

Like GATA-3, Itm2a is also preferentially expressed in cells of the T lineage among hematopoietic cells according to the Immgen database (http://www.immgen.org). Thus far, there are only a handful of published studies providing clues regarding the role of Itm2a in T cells. Itm2a was identified as a gene that was induced by MHC-mediated positive selection of double positive (DP) thymocytes [19]. Peripheral naïve Th and Tc cells expressed a low level of Itm2a, which was transiently induced when T cells were stimulated with concanavalin A. Three recent transcription profile studies independently found that the expression of Itm2a was significantly lower in Treg cells than in effector Th cells [20]-[22]. Marson et al further identified Itm2a as one of the most down-regulated genes in Treg cells and suggested that its expression was suppressed by Foxp3, the master transcription factor of Treg cells [21]. In addition, Itm2a was one of approximately 15 genes including CD3, Lck, and TCRβ, preferentially expressed in pediatric T-ALL [23]. Lately, Itm2a was linked to Graves' disease in a staged genome-wide association study [24], further strengthening its role in the immune system.

In this report, we show that the expression of Itm2a was reduced in GATA-3-deficient thymocytes and that Itm2a is transactivated by GATA-3. Although polyclonal thymocytes of Itm2a-deficient mice develop normally, Itm2a-deficient mice display a partial defect in the development of thymocytes bearing the MHC class I-restricted OT-I TCR but not the MHC class II-restricted OT-II TCR. Surprisingly, deficiency of Itm2a has little impact on the differentiation, activation, and function of T cells in vitro. However, Itm2a KO mice are not as efficient as wild type mice in mounting humoral immune responses against a model Th cell-dependent antigen. We further show that Itm2b was also expressed in T cells and very likely compensated for the loss of Itm2a.

## Materials and Methods

### Mice

The targeting construct of Itm2a was created through a recombineering-based method. The detailed strategy will be provided upon request. The targeting construct was then introduced into 129xB6 hybrid ES cells by inGenious Targeting Laboratory (Stony Brook, NY) to create floxed Itm2a mice. The *Itm2a* gene in the floxed allele was then deleted with EIIa-cre (The Jackson Laboratories) to create Itm2a-deficient mice, which were backcrossed to C57BL/6 mice for five generations before use. OT-I and OT-II mice (The Jackson Laboratories) were also crossed with Itm2a-deficient mice. Male or female mice aged 4–12 weeks were used. In all experiments, littermates were used as controls.

### Ethics statement

All animals were housed under specific pathogen-free conditions, and experiments were performed in accordance with the institutional guidelines for animal care under a protocol (#07-017) approved by the Institutional Animal Care and Use Committee (IACUC) of Dana-Farber Cancer Institute.

### Activation of T cells and in vitro differentiation of Th cells

Total cells were collected from spleens and activated with plate bound anti-CD3 (ranging from 0.5 µg/ml to 1 µg/ml), OT-II peptide (OVA 323-339), or OT-I peptide (SIINFEKL, OVA 257-264) at different concentrations. After 3 days, cells were collected and stained with surface markers. The supernatant was collected for cytokine analysis by ELISA. For in vitro differentiation of Th cells, CD4^+^ T cells were purified from spleens and lymph nodes by magnetic cell sorting (Miltenyi Biotec). The cells were stimulated with 1 µg/ml plate-bound anti-CD3 and 2 µg/ml soluble anti-CD28 under Th1 (3 ng/ml IL-12 plus 10 µg/ml anti-IL-4) or Th2 (10 ng/ml IL-4 plus 10 µg/ml anti-IFN-γ) conditions. For Th17 differentiation, total CD4^+^ cells were cultured in the presence of WT irradiated splenocytes (1∶3 ratio) and 3 ng/ml TGF-β1 and 20 ng/ml IL-6. Recombinant human IL-2 (100 U/ml) was added after 24 h, and the cells were expanded in complete medium containing IL-2. On day 7, the cells were restimulated with 50 ng/ml PMA and 1 µM ionomycin. The cytokine production was examined by intracellular cytokine staining.

### FACS analysis and antibodies

The following clones of antibody were purchased from Biolegend and used for cell surface staining: CD4 (RM4-5), CD8 (53-6.7), TCRβ (H57-597), B220 (RA3-6B2), CD69 (H1.2F3), CD25 (PC61), Vβ5 (MR9-4), IgM (RMM-1), IgD (11-26c.2a), CD21 (7E9), CD23 (B3B4). Flow cytometry was performed on a FACSCanto or FACSCanto II and analyzed with FlowJo software.

### Western blot and antibodies

In each sample, 1×10^6^ CD4^+^ cells were lysed in freshly prepared radioimmumoprecipitation assay buffer. Cell lysate was separated from debris by centrifugation at 12,000 rpm for 10 min. Lysate was loaded onto 12% polyacrylamide gels and transferred onto PVDF membrane (Polyscreen; Perkin Elmer). The membrane was subsequently blocked in 5% milk and probed with Itm2a antibody. The rabbit anti-human Itm2a antibody was generated by using Itm2a extracellular domain as an immunogen. Proteins were visualized using an ECL kit (PerkinElmer).

### Quantitative RNA analysis

Total RNA was purified using a Trizol Plus kit (Invitrogen). First-strand cDNA synthesis was performed on 200ng of total RNA using the QuantiTect Reverse Transcription kit (QIAGEN). Gene expression levels were determined by real-time PCR analysis performed using the Brilliant SYBR Green QPCR kit according to the manufacturer's protocol (Stratagene) on a MX-3000P apparatus (Stratagene) using the following cycling conditions: denaturation at 95°C for 30 s, annealing at 56°C for 60 s, and extension at 72°C for 30 s. Primer sets were designed using the Primer3 web utility. Levels of mRNA were adjusted for differences in *actb* (encoding beta-actin) expression. The following primers are used: Itm2a 5′-CGCACTGTCCGAGCTCAAAT-3' and 5'-CATCTCCCAGATGAGCCATCC-3'; Itm2b 5'-GAAGGTGACGTTCAACTCGG-3' and 5'- CTCTGTCCAACCGGAACCAC-3'; Itm2c 5'-GTCGCCATCAAGGCTGATAAA-3' and 5'-AGGCCCATGAGCAATACAACC-3'; actb 5'-GGCTGTATTCCCCTCCATCG-3' and 5'-CCAGTTGGTAACAATGCCATGT-3'.

### ELISA

Sandwich ELISA was performed using the following monoclonal antibody pairs (BD Biosciences): anti–IL-2 (JES6-1A12)/biotin-anti–IL-2 (JES6-5H4), and anti–IFN-γ (R4-6A2)/biotin-anti–IFN-γ (XMG1.2).

### Plasmid, transfection, and luciferase assay

An approximately 2 kb genomic fragment containing +28 to -1964 in relation to the transcriptional start site of the murine *Itm2a* gene was amplified and cloned into the BglII site of the PGL2-Basic (Promega) to create Itm2a-Luc. The IL-13-Luc and pcDNA3-GATA3 were previous reported [25]. In all luciferase assays, murine M12 B cells were transfected with 10 µg of luciferase reporter, 5 µg of pcDNA3 vector, and 5 µg pTK-Renilla with BIORAD GENE PULSER II set at 280 V and 0.975 F. Luciferase activity was determined in duplicate with Dual-Luciferase Reporter Assay System (Promega, Madison, WI). The firefly luciferase activity obtained from each sample was normalized against the renilla luciferase activity from the same sample.

### CTL killing

CTL killing assay was adapted from a published protocol [26]. Briefly, total splenocytes from WT and KO mice were cultured with irradiated splenocytes from Balb/c (H-2^d^) mice for 7 days and viable cells were collected as effector cells. EL4 (H-2^b^) or P815 (H-2^d^) target cells were label with CellTrack Far Red DDAO-SE (Molecular Probes Eugene, OR) at 37°C for 10 min. Effector and target cells (1×10^5^) were mix at different ratio and incubated at 37°C, 5% CO2 in a humidified incubator for 4 hours. Cells were collected, fixed and stained with anti-activated caspase-3 antibody (BD Bioscience CA).

### In vivo immunization and Th recall response

Mice were immunized with 20 µg trinitrophenyl-conjugated keyhole limpet hemocyanin KLH (TNP-KLH) (Biosearch Technologies, Novato, CA) emulsified in complete Freund adjuvant (CFA) through a subcutaneous injection. Immunized mice were sacrificed two weeks later. For recall response, 1×10^6^ lymph node cells were stimulated with 20 mg/ml TNP-KLH for 3 days, supernatant was collected and cytokine production was examined by ELISA.

## Results and Discussion

### Itm2a is a target gene of GATA-3 in thymocytes

**Figure 1:**
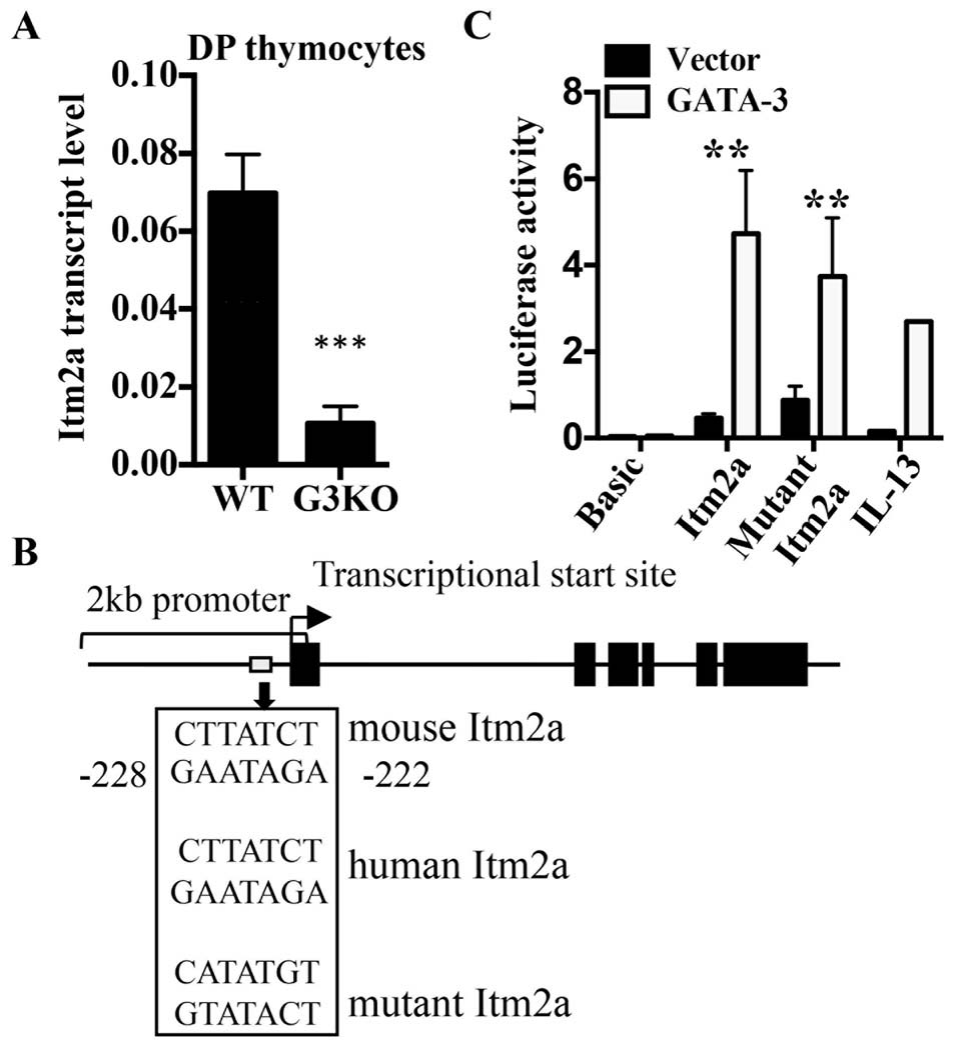
*Itm2a* is a target gene of GATA-3. **A**. Total RNA was prepared from DP thymocytes of WT and G3KO mice. The transcript level of *Itm2a* was quantified with real time PCR. The transcript level was then normalized against that of *Actb*. The data shown are means and standard deviation of three experiments. **B**. A schematic diagram of the 2 kb promoter of *Itm2a*. The location and sequence of the conserved and mutated GATA sites are shown. The black boxes represent exon of *Itm2a*. The thick arrow marks the transcription start site. **C**. M12 cells were transfected with the indicated luciferase reporter constructs along with a GATA-3 expression vector or an empty vector. The relative luciferase activity was calculated as described in the Materials and Methods. The data shown are means and standard deviations of three independent experiments.

To identify downstream targets of GATA-3 in thymocytes, we conducted gene chip analyses comparing the transcription profile between GATA-3-deficient (G3KO) and wild type DP thymocytes. We then focused on genes that were differentially expressed and have poorly characterized function. Among those genes, Itm2a was one of the most downregulated in G3KO DP cells. The differential expression of Itm2a was subsequently verified with real time PCR, confirming that Itm2a is a GATA-3 target gene in thymocytes (Figure 1A). We then examined the genomic sequence of mouse *Itm2a* with rVista 2.0 program (http://rvista.dcode.org) and found that the promoter region of *Itm2a* contains one consensus GATA binding site located at -222 to -228 relative to the transcriptional start site (Figure 1B). This site is also conserved in human *ITM2A* gene. In addition, overexpression of GATA-3 in M12 cells, which do not express GATA-3, transactivated a 2kb *Itm2a* promoter reporter, encompassing the conserved GATA site, as efficiently as an IL-13 promoter (Figure 1C), a known target of GATA-3. However, mutation of the putative GATA-3 site only modestly attenuated the transactivation by GATA-3 (Figure 1C), suggesting the presence of additional GATA sites. Overall, these data strongly suggest that Itm2a is a target of GATA-3 and provides an explanation for its T cell-specific expression among hematopoietic cells.

### Generation of Itm2a-deficient mice

**Figure 2:**
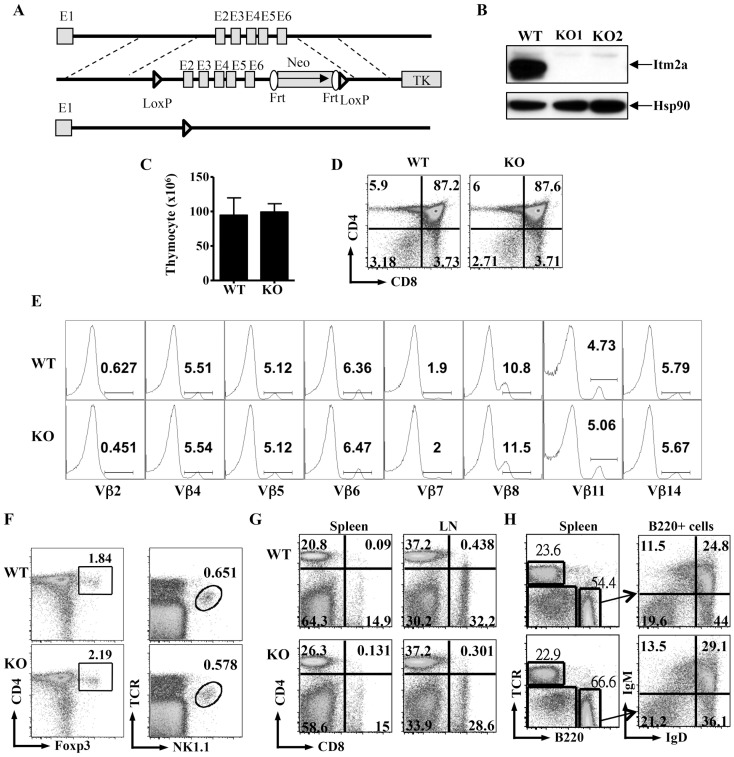
Deficiency of Itm2a has little impact on the differentiation of immune cells. **A**. Generation of Itm2a-deficient mice. Schematic diagrams of *Itm2a* gene (top), the targeting construct (middle), and deleted allele (bottom) are shown. Exons are represented with gray boxes and numbered. Regions for homologous recombination are marked with dashed lines. Neo and TK stand for neomycin cassette and thymidine kinase. **B**. T cells were purified from control (WT) and two Itm2aKO mice and stimulated with anti-CD3 for 72 hours. Cell extract from the stimulated cells was probed with anti-Itm2a and anti-Hsp90. **C**. Thymocytes of WT and Itm2aKO mice were enumerated, and the means and standard deviations are shown in the left panel. **D & E**. The thymocytes were also stained with anti-CD4/anti-CD8 (**D**) and anti- α/β TCR, and TCR^high^ cells were further stained with antibodies against various Vβ chains indicated in **E**. **F**. The thymocytes were stained with anti-CD4/anti-Foxp3 and anti-TCR/NK1.1. **G**. Splenocytes and lymph node cells of WT and Itm2aKO mice were stained with anti-CD4 and anti-CD8. **H**. The splenocytes were also stained with anti-TCR/anti-B220. B220^+^ cells were then further separated by anti-IgM/anti-IgD staining. The data shown in **D–H** are representative FACS plots of at least three independent experiments.

The expression of Itm2a is induced in activated T cells [19]. To investigate the function of Itm2a in T cells, we transduced activated Th cells with a retrovirus expressing Itm2a. However, we found that overexpression of Itm2a had little impact on the activation, survival, and cytokine production of Th cells (data not shown). Therefore we set out to generate Itm2a-deficient (Itm2aKO) mice. We inserted a neomycin cassette and two loxp sites into the *Itm2a* allele (Figure 2A). We then deleted the *Itm2a* gene in germline with the EII-a Cre transgene. Itm2aKO mice were born according to Mendelian ratios and had normal development and growth up to 6 months of age (data not shown). T cells obtained from Itm2aKO mice contained no detectable Itm2a protein (Figure 2B), confirming the complete deletion of the *Itm2a* gene. This lack of developmental defect is consistent with the results of a recent publication [27], in which an independently generated Itm2a-deficient mouse strain was studied.

### T cell development is normal in Itm2aKO mice

Although Itm2a transgenic mice have a subtle aberration in the expression of CD8 [19], we found that deficiency of Itm2a had no apparent impact on the development of thymocytes. The total number of thymocytes and the percentage of CD4 single positive (SP), CD8SP and CD4^+^ CD8^+^ cells were very comparable between WT and Itm2aKO mice (Figure 2C and 2D). In addition, the mean fluorescence intensity of CD4, CD8, HSA, TCR, and Qa2 was normal in Itm2aKO thymocytes (data not shown). We further investigated the TCR repertoire with antibodies against various Vβ chains and found no apparent difference between WT and Itm2aKO mice (Figure 2E). The numbers of Foxp3^+^ Treg cells and natural killer T (NKT) cells were also normal (Figure 2F). Similarly, there was no detectable difference in the number and CD4/CD8 ratio of T cells in peripheral lymphoid organs (Figure 2G). Furthermore, the development and expression of various differentiation markers of other immune cells including B cells and myeloid cells were unaltered in the absence of Itm2a (Figure 2H and data not shown).

### Normal activation of Itm2aKO T cells

**Figure 3:**
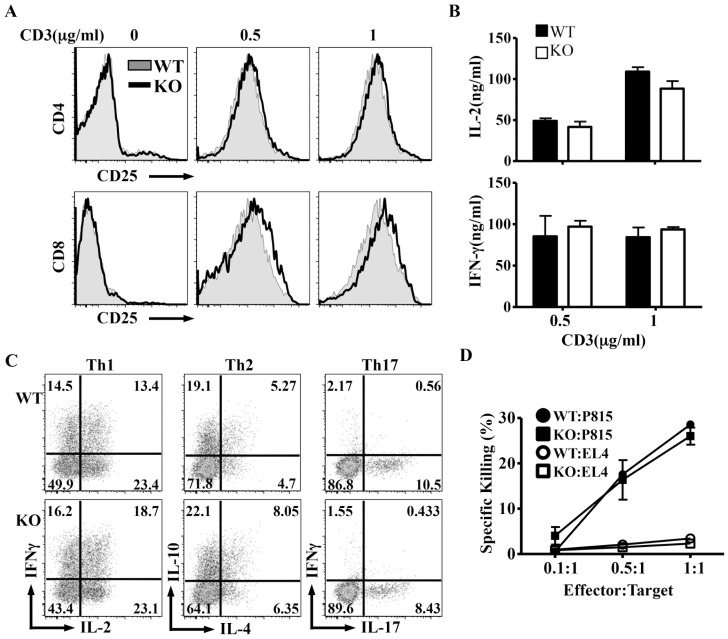
Deficiency of Itm2a has no impact on the function of peripheral T cells. **A & B**. Splenocytes of WT and Itm2aKO mice were stimulated with anti-CD28 (2 µg/ml) and indicated amount of anti-CD3 for three days. The expression of CD25 by CD4^+^ and CD8^+^ T cells was examined with FACS and is shown in **A**. The concentration of IL-2 and IFN-γ in the supernatant of stimulated cells was quantified with ELISA. The means and standard deviations of three independent experiments are shown in **B. C**. Naive Th cells of WT and Itm2aKO mice were differentiated in vitro under Th1, Th2, and Th17 polarization conditions, and re-stimulated with PMA/ionomycin. The production of indicated cytokines was examined with intracellular cytokine staining. Representative FACS plots are shown. **D**. CTL activity of WT and Itm2aKO CD8^+^ T cells was examined according to the protocol described in the *Material and Methods*. The data shown are means and standard deviations of three independent experiments.

We then examined if loss of Itm2a would affect T cell activation. We stimulated splenocytes with escalating doses of anti-CD3 for 3 days. The induction of activation markers including CD25 and CD69, and cell recovery were comparable between WT and Itm2aKO CD4^+^ and CD8^+^ T cells (Figure 3A and data not shown). There was also no difference in the production of IL-2 and IFN-γ (Figure 3B). In addition, Itm2aKO Th cells differentiated normally into Th1, Th2, and Th17 cells in vitro (Figure 3C). We also examined CTL activity against MHC mismatched targets of Itm2aKO CD8^+^ T cells and found no apparent defect (Figure 3D). Taken all together, Itm2a was dispensable for the development and function of polyclonal T cells.

### Itm2a regulates the development of OT-I but not OT-II thymocytes

**Figure 4:**
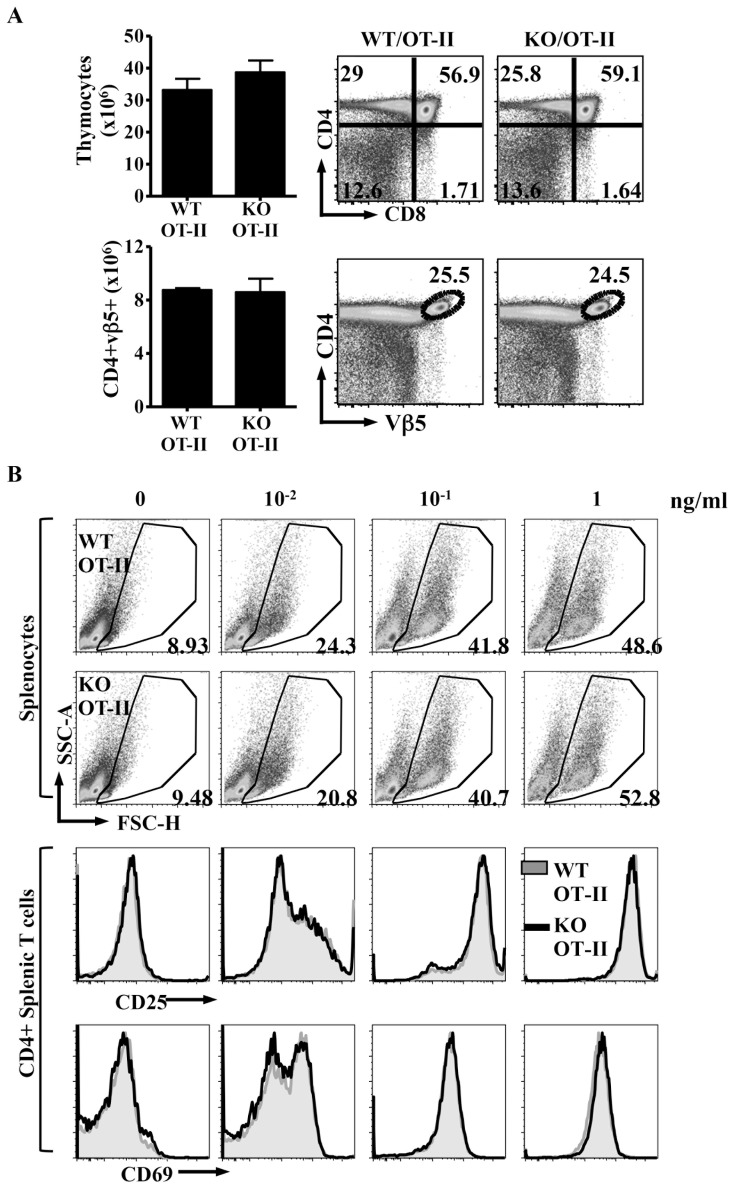
Deficiency of Itm2a does not affect the selection and activation of OT-II T cells. **A**. Thymocytes of WT/OT-II and KO/OT-II mice were stained with anti-CD4, anti-CD8, and anti-Vβ5. Representative FACS plots are shown. The means and standard deviations of total thymocytes and CD4^+^Vβ5^+^ thymocytes from three experiments are shown in the left panels. **B**. Splenocytes of WT/OT-II and KO/OT-II mice were stimulated with ovalbumin peptides at indicated concentration for 3 days. The percentages of live cells were determined based on the FSC/SSC gate and are shown. The cells were also stained with anti-CD25 and anti-CD69. Representative FACS plots are shown.

To address whether Itm2a deficiency might affect the development of monoclonal thymocytes, we first crossed Itm2aKO mice with OT-II TCR transgenic mice, which bear an MHC class II-restricted TCR recognizing a peptide derived from ovalbumin. Introduction of the OT-II TCR comparably increased the percentage and number of CD4SP thymocytes cells in both WT and Itm2aKO mice (Figure 4A, upper row). The number of mature OT-II CD4^+^ thymocytes was normal in the absence of Itm2a (Figure 4A, lower row). We further stimulated WT/OT-II and Itm2aKO/OT-II splenocytes with the cognate ovalbumin peptide and found no difference in the expansion and activation of OT-II CD4^+^T cells (Figure 4B). Thus, deficiency of Itm2a very likely does not affect MHC class II-mediated selection and antigen-specific activation of CD4^+^ T cells.

**Figure 5:**
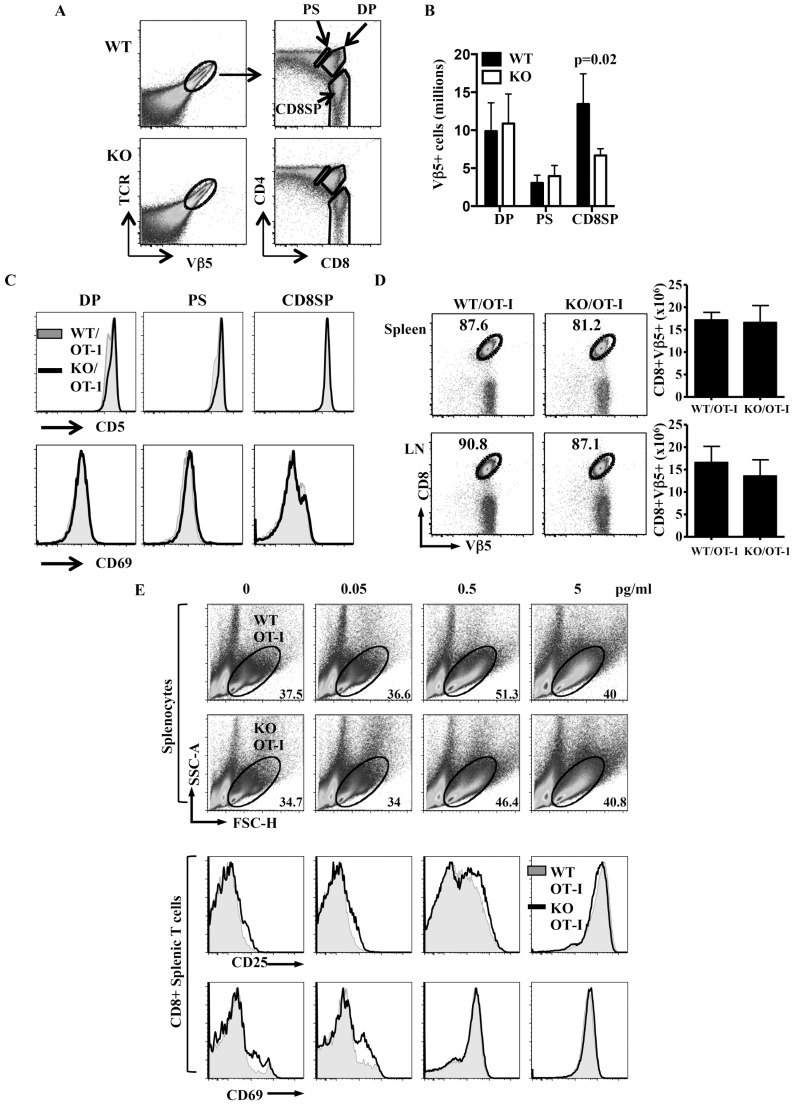
Itm2a regulates the thymic development but not the activation of OT-I cells. **A**. Thymocytes of WT/OT-I and KO/OT-I mice were stained with anti-Vβ5, anti-α/β TCR, anti-CD4, and anti-CD8, and. representative TCR/Vβ5 FACS plots are shown in the left column. The CD4/CD8 FACS plots of TCR^+^Vβ5^+^ cells are shown in the right column. The DP, post-selected CD4+CD8dull (PS), and CD8SP populations of TCR^+^Vβ5^+^ cells are gated. B. The numbers of DP, PS, and CD8SP Vβ5^+^ cells were enumerated from three pairs of mice. The means and standard deviations are shown. Statistical analysis was performed with Student's t tests. **C**. The histograms of CD69 and CD5 of indicated Vβ5^+^ thymocytes of WT/OT-I and KO/OT-I mice are overlaid and shown. D. Splenocytes and lymph nodes cells of WT/OT-I and KO/OT-I mice were stained with anti-Vβ5 and anti-CD8. Representative CD8/Vβ5 FACS plots and the absolute numbers of CD8^+^Vβ5^+^ cells are shown. **E**. Splenocytes of WT/OT-I and KO/OT-I mice were stimulated with ovalbumin peptides at the indicated concentrations for 3 days. The percentages of live cells were determined based on the FSC/SSC gate and are shown. The cells were also stained with anti-CD25 and anti-CD69. Representative FACS plots are shown.

We then introduced the OT-I TCR transgene, a MHC class I-restricted TCR, into Itm2aKO mice. In OT-I mice, nearly all TCR^+^ cells are also positive for Vβ5 (Figure 5A). Interestingly, we found that deficiency of Itm2a led to an approximately 50% reduction in the number of mature Vβ5^+^CD8^+^SP OT-I thymocytes (Figure 5A and 5B). One possible explanation for the reduction in the number of mature OT-I thymocytes in the absence of Itm2a is that Itm2a is required for the optimal positive selection. Upon positive selection by MHC, DP thymocytes up-regulate CD69 and CD5, and become CD4^+^CD8^dull^ before transiting into SP cells. However, the induction/expression of CD69 and CD5 in DP and post-selected CD4^+^CD8^dull^ Vβ5^+^ thymocytes was very comparable between WT/OT-I and Itm2aKO/OT-I mice (Figure 5C). In addition, the absolute number of Vβ5^+^ OT-I cells was normal in the absence of Itm2a until the CD8SP stage when an approximately 50% reduction in the number was observed (Figure 5B). This data strongly suggest that deficiency of Itm2a has little impact on MHC-mediated selection of OT-I cells and that Itm2a deficiency results in a partial defect in the transition from CD4^+^CD8^dull^ to CD8SP stage. The cause of this defect is still unclear but is unlikely due to enhanced apoptosis of CD8SP cells because there was no apparent difference in the level of Annexin V and 7-AAD between WT/OT-I and Itm2aKO/OT-I CD8SP thymocytes (data not shown)

Despite the reduction in number, the subsequent maturation of the Itm2aKO/OT-I CD8SP thymocytes appeared to be normal based on the down-regulation of HSA and the induction of Qa2 (data not shown). In addition, deficiency of Itm2a did not affect the number of OT-I T cells in peripheral lymphoid organs (Figure 5D); and stimulation of WT and Itm2aKO OT-I splenocytes with the cognate peptide resulted in comparable cell expansion and induction of activation markers (Figure 5E).

### Attenuated T cell-dependent humoral responses in Itm2aKO mice

**Figure 6:**
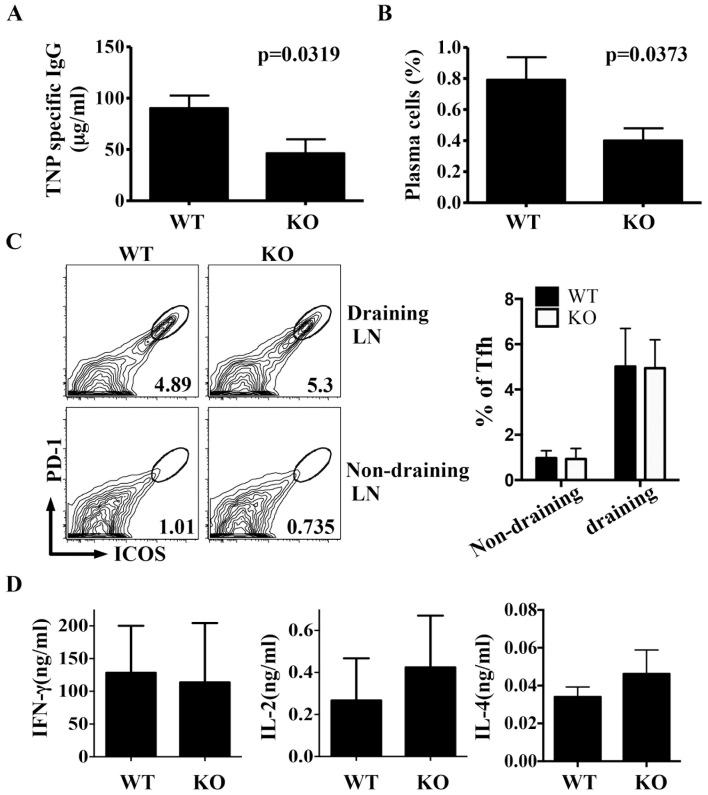
Impaired immune response to Th cell-dependent antigens in the absence of Itm2a. WT (N = 8) and Itm2aKO (N = 7) mice were immunized with TNP-KLH in CFA at tail base. Two weeks after the immunization, the level of TNP-specific IgG was quantified with ELISA (**A**). The percentage of plasma cells (CD138^+^B220^dull^) among splenocytes was calculated (**B**). T cells of draining (inguinal) and non-draining (axillary) lymph nodes were further stained with anti-ICOS/anti-PD-1. ICOS^+^PD-1^+^ Tfh cells were gated (**C**). The means and standard deviations of the percentage of Tfh among lymph node Th cells are shown in the right panel of **C**. Draining LN cells were restimulated with TNP-KLH for 24 hours. The concentration of IFN-γ and IL-2 in the supernatant of the stimulated cells was quantified with ELISA (D). Statistical analyses were performed with unpaired Student's t tests.

We subsequently asked whether lack of Itm2a would affect T cell-dependent immune responses in vivo. We immunized mice with CFA-emulsified TNP-KLH and found that immunized Itm2aKO mice had less TNP-specific IgG in serum compared to control mice (Figure 6A). Immunized Itm2aKO mice also had fewer plasma cells in their spleens (Figure 6B). However, the number of follicular helper T (Tfh) cells was normal in Itm2aKO mice (Figure 6C). We then restimulated lymph node cells obtained from immunized mice with TNP-KLH in vitro and found that the production of IL-2 and IFN-γ by the restimulated lymph node cells was not affected by the absence of Itm2a (Figure 6D). It is unclear how deficiency of Itm2a regulates the homeostasis of plasma cells and the production of antigen-specific IgG. Given the preferential expression of Itm2a in T cells, these defects are very likely intrinsic to T cells. However, we cannot rule out the possibility that Itm2a has a B cell-intrinsic role. Examining mice deficient in Itm2a only in T or B cells will clarify this issue.

### Possible functional redundancy among the members of the itm2 family

**Figure 7:**
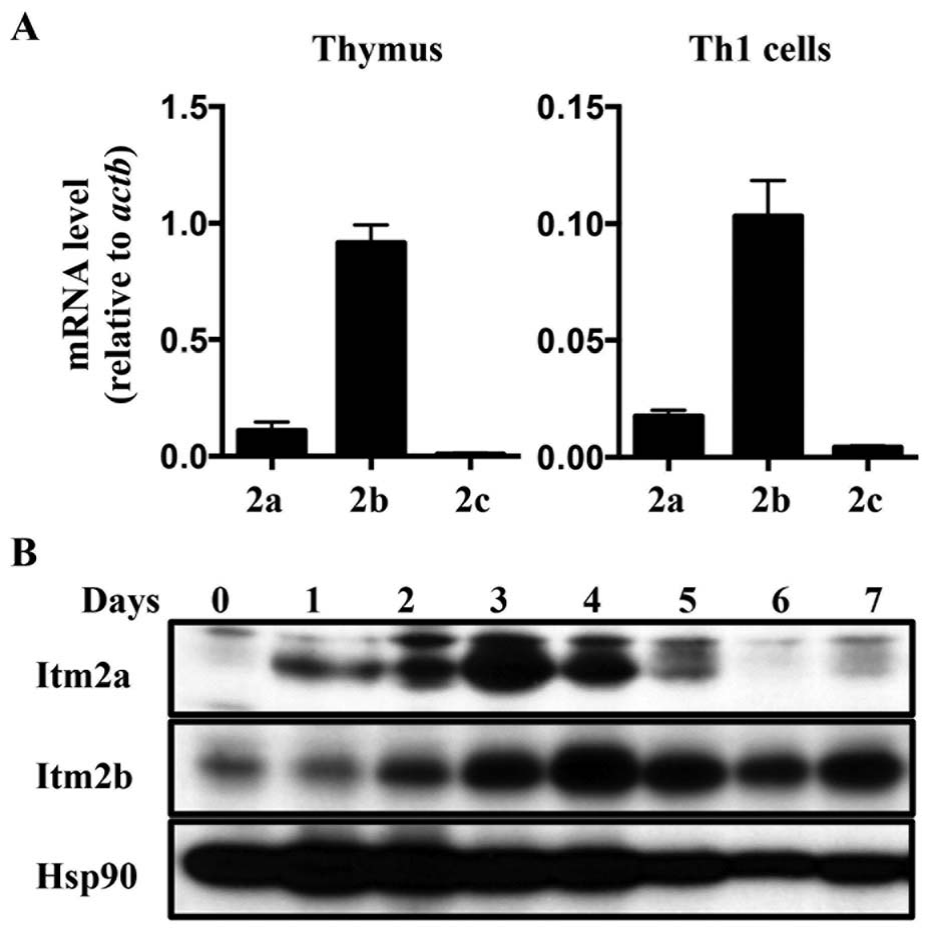
Potential functional redundancy between Itm2a and Itm2b. **A**. The transcript level of *Itm2a*, *Itm2b*, and *Itm2c* in WT thymocytes and WT Th1 cells were quantified with real time PCR, and normalized against that of *Actb*. The means and standard deviations of three experiments are shown. **B**. WT naive Th cells were stimulated in vitro with anti-CD3 (1 µg/ml)/anti-CD28 (2 µg/ml). Whole cell extract was harvested at indicated time points and probed with indicated antibodies. The data shown are representative of two independent experiments.

Our data indicate that Itm2a is a target gene of GATA-3. However, the phenotype of Itm2a deficiency was rather modest. This modest phenotype raises the possibility that Itm2b and/or Itm2c may compensate for the loss of Itm2a. We therefore compared the expression of these three genes in thymocytes and activated Th cells. We found that the transcript level of *Itm2b* was almost 10 times higher than that of *Itm2a* in thymus and activated T cells (Figure 7A). In contrast, the transcript of *Itm2c* was almost undetectable. In agreement with the published data, the protein level of Itm2a gradually increased in response to in vitro stimulation and peaked at day 2 after stimulation (Figure 7B), but quickly faded away after day 4. The level of Itm2b protein also increased in activated T cells but peaked at day 3-4. Unlike Itm2a, the level of Itm2b was maintained up to seven days after stimulation. This result suggests that Itm2b very likely compensates for the loss of Itm2a. Itm2b-deficient mice have been generated, but their immune phenotype has never been reported. It will of great interest to examine the development and function of T cells in mice deficient in Itm2b or both Itm2a and Itm2b.

In summary, *Itm2a* is a target gene of GATA-3 in thymocytes. It is required for the optimal development of OT-I thymocytes and humoral responses to Th cell-dependent antigens. However, its role in the adaptive immunity is rather modest probably due to functional compensation from Itm2b, which is also highly expressed in T cells. Thus, the combined function of Itm proteins remains to be determined.
